# Pathologic complete response following low-dose radiation for advanced oral cavity cancer in a patient with human immunodeficiency virus

**DOI:** 10.1186/s40463-022-00586-6

**Published:** 2022-10-04

**Authors:** Amy B. Leming, Andrea L. Johnston, Greg A. Krempl, Evan J. Fowle, Daniel J. Morton, Christina E. Henson

**Affiliations:** 1 College of Medicine, University of Oklahoma Health Sciences Center, Oklahoma City, OK USA; 2 Department of Radiation Oncology, University of Oklahoma Health Sciences Center, 800 NE 10th Street, L100, Oklahoma City, OK 73104 USA; 3 Department of Otorhinolaryngology, University of Oklahoma Health Sciences Center, Oklahoma City, OK USA; 4 Stephenson Cancer Center, University of Oklahoma Health Sciences Center, Oklahoma City, OK USA; 5 Department of Pathology, University of Oklahoma Health Sciences Center, Oklahoma City, OK USA; 6 Department of Pediatrics, University of Oklahoma Health Sciences Center, Oklahoma City, OK USA; 7 Present Address: Department of Otorhinolaryngology – Head and Neck Surgery, The University of Texas Health Science Center at Houston, Houston, TX USA

**Keywords:** Oral cavity cancer, Radiation therapy, HIV, HAART

## Abstract

**Background:**

Advanced squamous cell carcinoma (SCCa) of the oral cavity is often not amenable to curative-intent therapy due to tumor location, tumor size, or comorbidities.

**Case presentation:**

A 51-year-old male patient with human immunodeficiency virus and on highly active antiretroviral therapy (HAART) presented with a cT4aN2c SCCa of the tongue. He received a preoperative single course of Quad-Shot radiation therapy to 14 Gy in 4 fractions followed by surgical resection. Patient had no residual carcinoma on surgical pathology and no evidence of disease on subsequent clinical and radiological exams.

**Conclusions:**

To our knowledge, this is the first case of pathologic complete response for a patient on HAART following a single cycle of the Quad-Shot regimen for advanced oral cavity SCCa. Protease inhibitors in HAART can induce spontaneous tumor regression via inhibition of proteasome function and activation of apoptosis, and thus act as a cancer therapeutic.

**Graphical Abstract:**

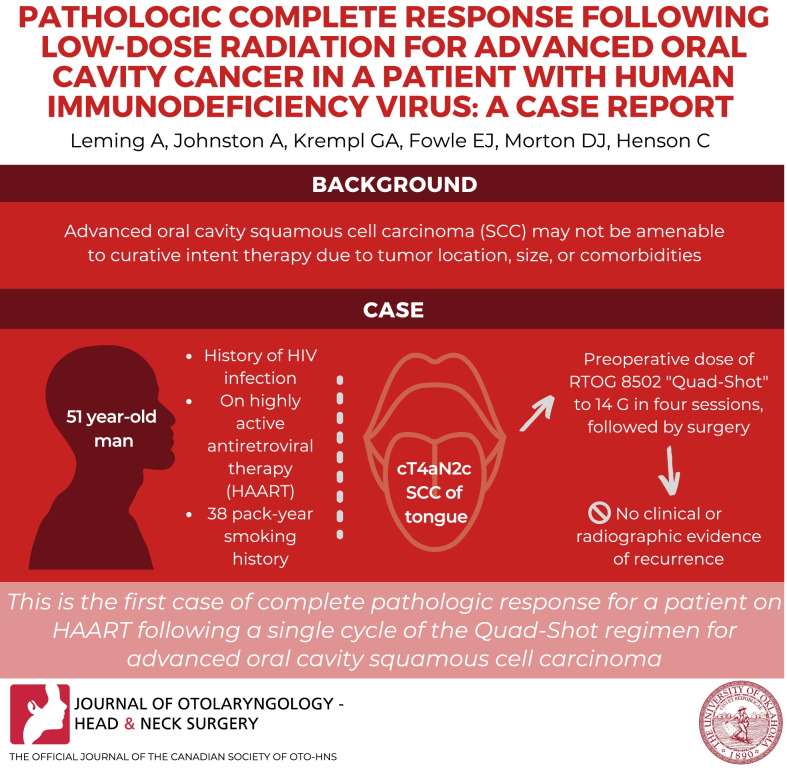

## Background

Oral cavity cancer comprises approximately 2% of all new cancer diagnoses in the United States, with squamous cell carcinoma (SCCa) accounting for the majority of cases [1–3]. The oral tongue is the most common subsite for oral cavity SCCa [1, 3], which historically affects older, male adults with exposure to well-established risk factors such as tobacco and alcohol use; however, recent publications report an increasing incidence of oral cavity SCCs, particularly in younger, non-smoker, female patients [4–7]. At the time of diagnosis, approximately 40% of patients have advanced-stage lesions (stages III-IV) [2].

Treatment of advanced disease begins with surgical resection of the primary tumor, often with neck dissection and reconstruction. According to current NCCN guidelines, adjuvant postoperative treatment with chemotherapy and or radiation therapy (RT) is indicated for tumors with advanced features and therefore at high risk of locoregional recurrence [8]. This includes patients with large primary tumors (pT3 or pT4), bulky nodal disease (pN2 or pN3), metastases to nodal levels IV or V, positive surgical margins, lymphovascular invasion, perineural invasion, and extracapsular spread [8]. However, many cases are not amenable to curative-intent therapy due to tumor location, tumor size, or comorbidities; hypofractionated RT is used to palliate symptoms and improve quality of life in these circumstances [9].

In a recent systematic review, Graboyes et al. suggest that treatment delays after diagnosis can negatively impact survival, with the suggestion that 20 or fewer days between diagnosis and initiation of treatment is ideal [10]. For large tumors that require complex reconstruction with a team of surgeons, this can be difficult to achieve, and there is interest in “window of opportunity” trials with agents such as PDL1 inhibitors. However, there is concern that the timing and side effects associated with systemic therapy may prohibit surgery; an alternative option is to employ neoadjuvant RT. Herein we report our experience with the first case of oral cavity SCCa treated with a single cycle of neoadjuvant hypofractionated RT resulting in a pathologic complete response (pCR).

## Case presentation

**Fig. 1:**
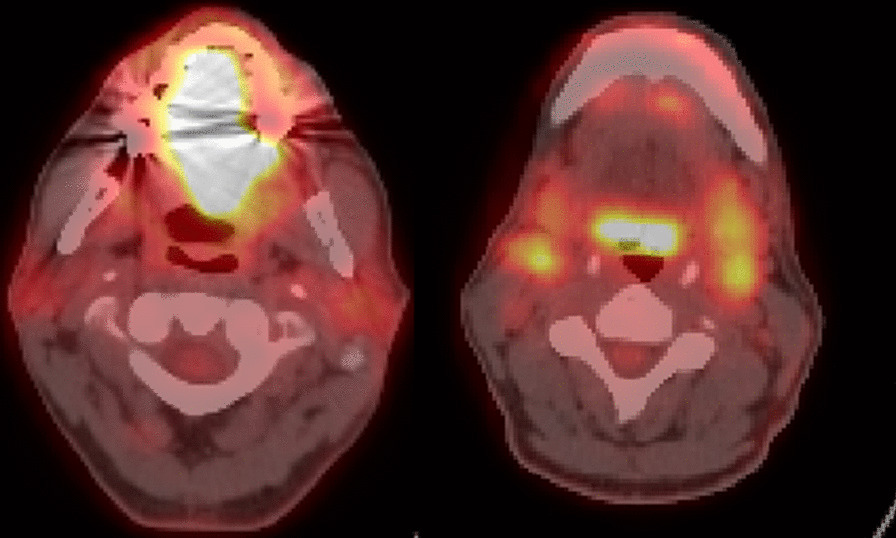
PET/CT at diagnosis showing large, hypermetabolic tongue mass (left image) and avid lymph nodes (right image)

**Fig. 2:**
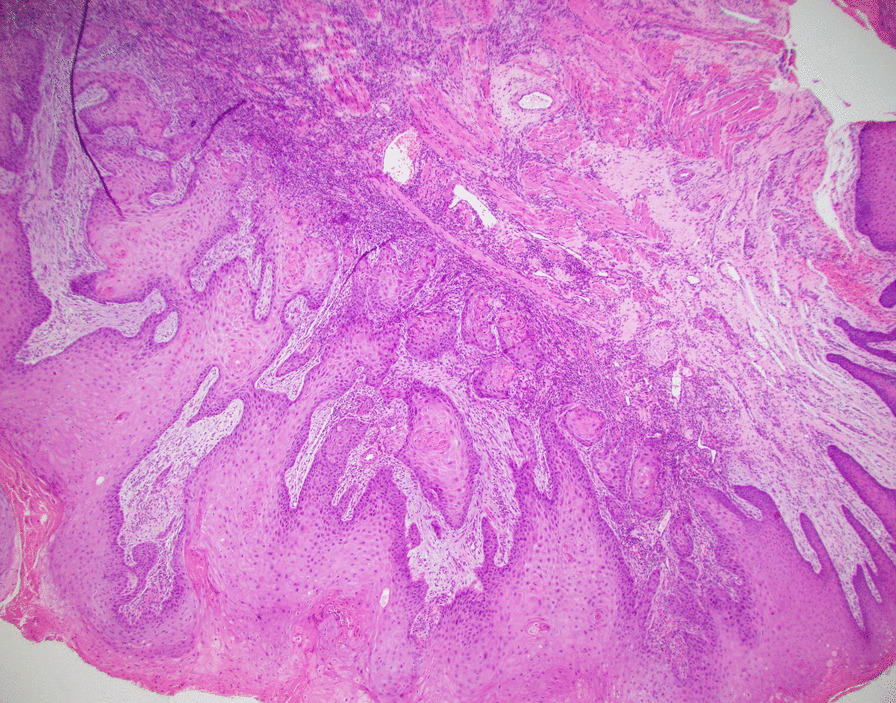
Pretreatment tongue biopsy; H&E 4x: Invasive well-differentiated squamous cell carcinoma, keratinizing subtype invading to a depth of 2 mm

A 51-year-old man with history of human immunodeficiency virus (HIV) infection, highly active antiretroviral therapy (HAART), and 38 pack-years smoking presented with an enlarging tongue mass. Exam noted a 4.5 × 1.9 cm ulcerated tongue lesion that had been growing for seven months prior to presentation. Imaging supported the physical exam with a PET/CT demonstrating an ill-defined hypermetabolism throughout the tongue with additional FDG-avid lingual tonsils, bilateral level 2A and 3 lymph nodes, and bilateral inguinal lymph nodes (Fig. 1). Incisional biopsy of the tongue mass was positive for invasive SCCa, keratinizing, and well-differentiated with a depth of 2 mm (Fig. 2). The cancer was staged cT4a N2c M0, Stage IVA (AJCC 8th edition) by our institutional multidisciplinary head and neck tumor board, based on maximum tumor size of 6 cm on clinical exam and PET finding of diffuse hypermetabolism throughout the entirety of the tongue with standardized uptake value (SUV) of 17, leading to assignation of clinical depth of invasion of > 10 mm. The recommended treatment was surgery followed by appropriate adjuvant therapy based on final pathology.

**Fig. 3:**
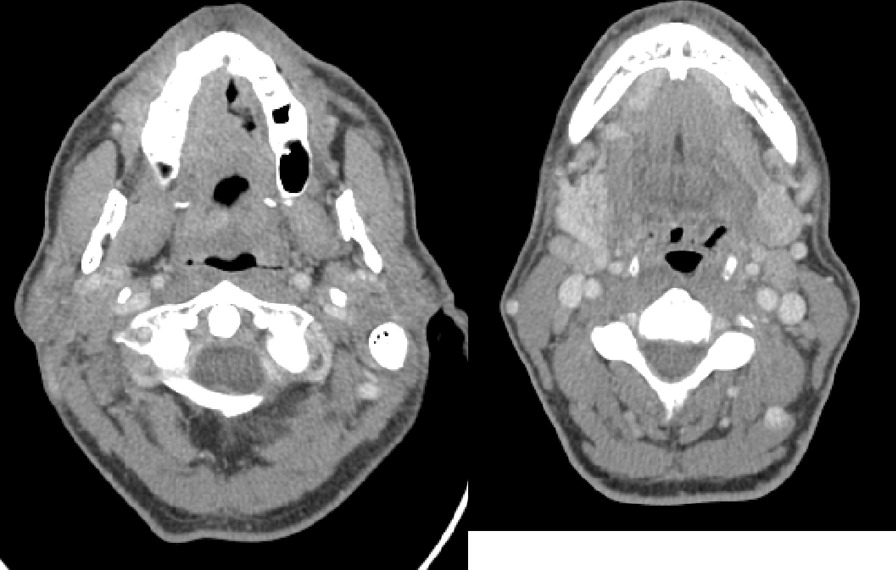
Post-radiation CT images with contrast

The patient underwent neoadjuvant hypofractionated radiation therapy as a temporizing measure while awaiting surgery which was delayed due to social and scheduling factors. He was treated with one course of the RTOG 8502 “Quad-Shot” regimen to a dose of 14 Gy given in four fractions over the course of two days to gross disease (primary tumor and involved nodes). Repeat CT neck immediately prior to surgery showed an increase in size of the dorsal tongue lesion, with re-demonstration of prominent right-sided cervical lymph nodes, now also increased in size (Fig. 3).

The patient underwent subtotal glossectomy and bilateral selective neck dissection with pectoralis muscle reconstruction eight weeks after completion of RT. The operative report describes a mucosalized ulcerative lesion involving the anterior two-thirds of the tongue. Final pathology demonstrated no residual carcinoma, 0/28 left cervical lymph nodes positive for carcinoma, and 0/30 right cervical LN positive for carcinoma. At one month follow up he was reported to be doing well overall, with expected dysphagia related to surgical changes. Physical exam revealed no evidence of disease. CT neck 2.5 months following surgery, four months following Quad-Shot RT, showed no evidence of disease. His case was again discussed at multidisciplinary tumor board, with a new group consensus to refrain from adjuvant therapy. Unfortunately, he was subsequently lost to follow-up.

**Fig. 4:**
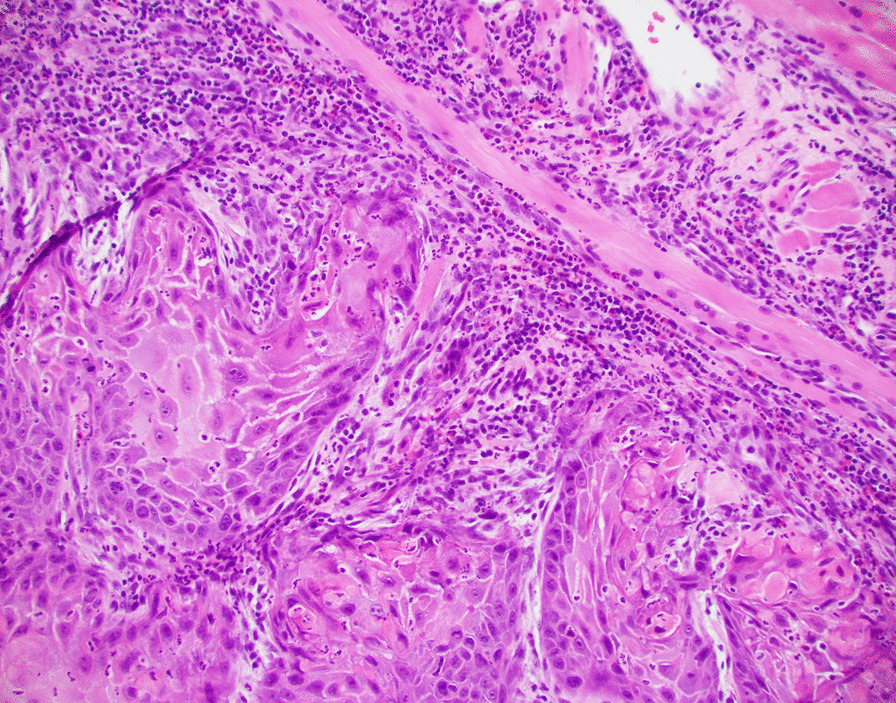
Pretreatment tongue biopsy; H&E 20x: Nests of infiltrative malignant squamous cells in a background of desmoplastic stroma and mixed inflammation

**Fig. 5:**
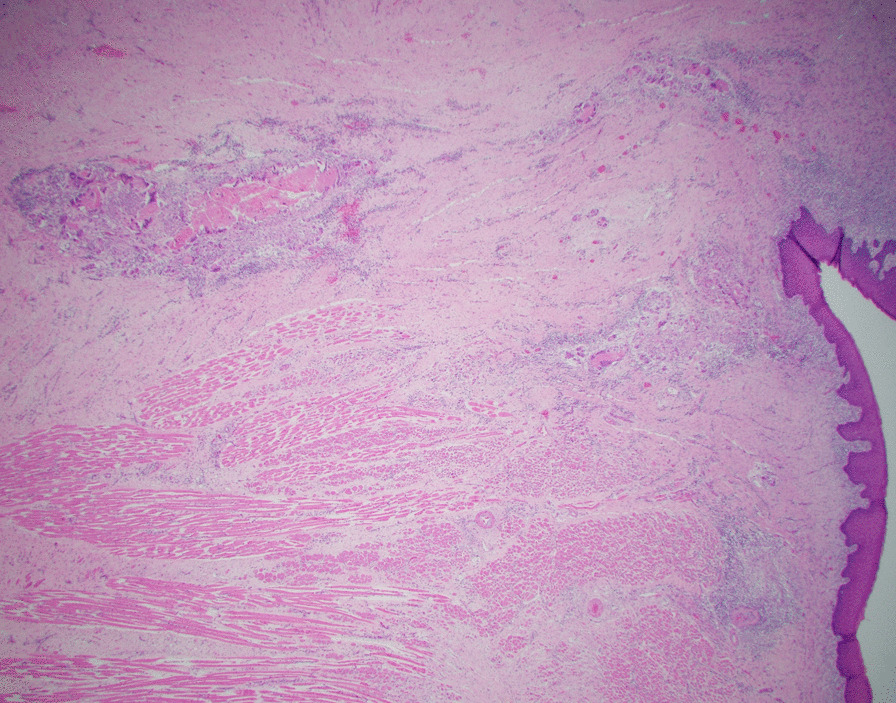
Post-treatment tongue resection; H&E 2x: Benign overlying squamous mucosa with keratin granulomas to a depth of 7 mm

**Fig. 6:**
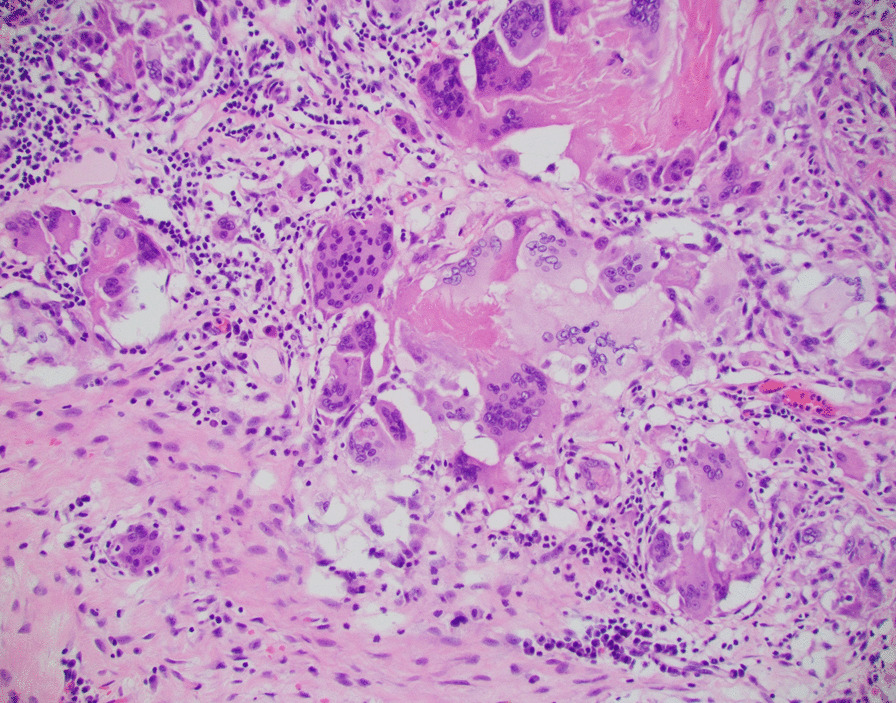
Post-treatment tongue resection; H&E 20x: Keratin granuloma comprised of multinucleated giant cells arranged around anucelate keratin debris; consistent with complete pathologic response to treatment

**Fig. 7:**
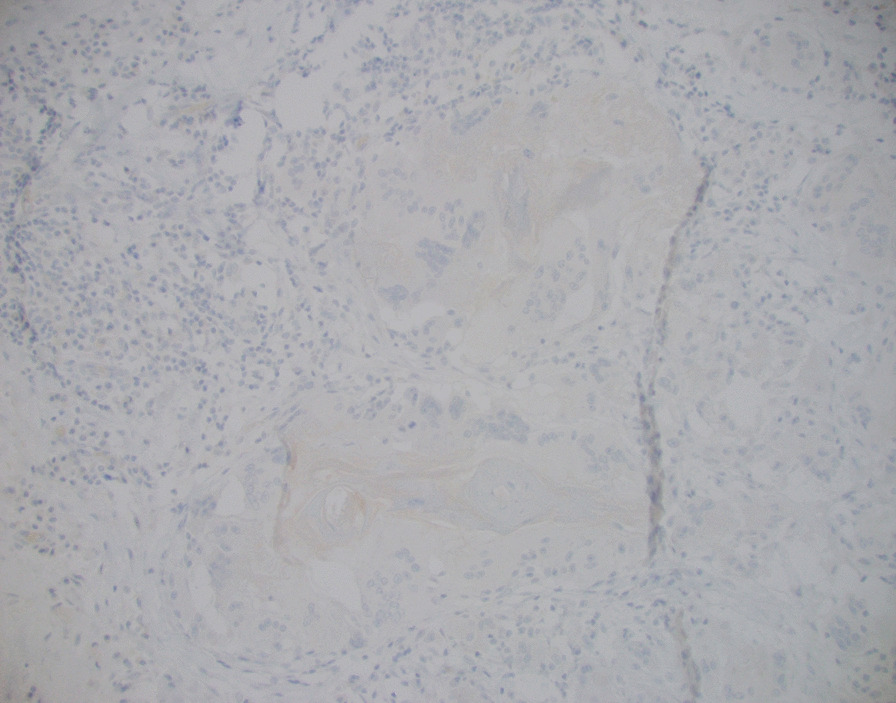
Post-treatment tongue resection; p40 IHC 20x: Negative p40 immunohistochemical stain confirming no viable squamous cells

The patient’s initial biopsy and surgical specimen were reviewed after the fact to confirm the initial cancer diagnosis and the pCR. Review of initial biopsy confirmed at least 2 mm depth of SCCa (Figs. 2 and 4). Review of the surgical pathology glossectomy specimen confirmed no residual disease, with residual keratin granulomas which were 7 mm deep, suggesting at least a commensurate depth of invasion of the tumor prior to treatment (Figs. 5, 6 and 7). Of note, the neck dissection was without evidence of treatment related changes.

## Discussion

Here we describe one patient with advanced stage oral cavity SCCa who had complete pathologic response following one course, 14 Gy, of the Quad-Shot regimen employed neoadjuvantly while the patient was awaiting surgery. Radiographically, he appeared to have progressed after neoadjuvant therapy, both at the primary site and the regional lymphatics. His cervical adenopathy was considered pathologic based upon likelihood for metastasis, clinical appearance/feel, and pre-treatment PET avidity. Because his advanced primary site would require treatment of regional lymphatics (neck dissection for surgical patients), it was not deemed necessary to biopsy the enlarged nodes at presentation. It is possible that the initial enlargement and radiographic progression of the lymph nodes was due to his underlying HIV disease rather than from his oral tongue cancer. Regardless of the initial status of the neck, a pCR was found at the primary site.

For this patient, consideration has to be given to the potential of spontaneous tumor regression and the additional benefit of HAART in his care. This patient’s HAART regimen consisted of darunavir, dolutegravir, and tenofovir. Spontaneous tumor regression is not a new phenomenon and has been reported in about one in 60,000–100,000 malignancies [11]. Melanoma, renal cell carcinoma, and neuroblastoma are among the most common cancers associated with spontaneous regression, although SCCa has also been reported. The exact cause of tumor regression is not known; there is extensive speculation with one possible explanation being activation of the immune system [12]. The innate immune system is believed to play a role in spontaneous tumor regression, as the phenomenon has been observed following acute infections in which the patient experiences fever due to strong activation of the immune system [13]. Indeed, there are ongoing clinical trials evaluating the QUAD shot regimen in combination with immunotherapy in patients with head and neck cancer, including our own (NCT04373642).

Protease inhibitors are important in HAART therapy for HIV patients. Protease inhibitors, specifically saquinavir and ritonavir, reportedly have antitumor effects through inhibition of 26 s and 20 s proteasome function [14, 15]. The protease inhibitors inhibit the anti-apoptotic NF-KB transcription factor thereby inducing apoptosis. Prostate cancer cells treated with saquinavir show increased sensitivity to chemotherapy and RT [14]. A prospective study of HIV-seropositive women with cervical squamous intra-epithelial lesions (SIL) and on HAART therapy, showed a statistically significant improvement in the prevalence of SIL on Papanicolaou smears as well as on colposcopy [16]. Multiple phase I and phase II clinical trials of the protease inhibitor nelfinavir, chosen for its increased efficacy in preclinical settings, in combination with chemotherapy and RT are ongoing and show encouraging results for multiple types of unresectable solid tumors including liposarcoma, adenocarcinoma of the pancreas, and non-small cell lung carcinoma [17]. Nelfinavir induces apoptosis through inhibition of growth factor receptor activation and Akt signaling [18]. Our patient was on HAART before, during and after RT, including the protease inhibitor darunavir, and this HIV therapy may have contributed to the pCR demonstrated after low-dose RT.

The Quad-Shot regimen has displayed success in the palliative setting for many patients. The Quad-Shot hypofractionated palliative RT regimen was originally devised for advanced pelvic malignancies but has since been adapted for palliation in patients with incurable head and neck cancers [9, 19]. It is thought to be well suited to the head and neck setting, since it delivers a biologically equivalent dose just below the threshold for producing mucositis, and the interval of separation is sufficient for depleted mucosal stem cells to repopulate before the next cycle. The regimen, as designed, consists of 3.5 Gy delivered twice a day for two consecutive days and may be repeated every three or four weeks for a total dose of 42 Gy in three cycles with the goal of adequate palliation while limiting acute and late toxicity [9]. Studies show that with even one cycle of Quad-Shot, patients may achieve approximately 55% palliation, with almost 90% reduction in symptoms with 3 cycles [9, 20, 21]. Additionally, the tumor response rates and decreased toxicity with Quad-Shot are comparable to conventional hypofractionated palliative RT (30 Gy/10 fractions/2 weeks) [22].

Although surgery followed by concurrent RT and/or chemoradiotherapy is the management choice recommended by the NCCN guideline for treating advanced oral tongue squamous cell carcinoma, 5-year survival rates remain low at < 40% [23]. In addition, a preoperative pCR is an independent prognostic factor in advanced SCCa. Kirita et al. reported the long-term prognostic value of achieving pCR in such patients who were treated preoperatively with cisplatin- or carboplatin-based chemotherapy in combination with simultaneous irradiation (target dose 40 Gy), and showed that 10-year progression free survival of patients with pCR was significantly better than that of patients with residual tumor (87.5% vs. 40%) [24].

Although Quad-Shot is reportedly effective for palliation postoperatively or in unresectable disease, no study has evaluated its effectiveness in preoperative tumor control. There are potential advantages to this regimen, as there is a high tolerance with low toxicity, shortened treatment time, and excellent local tumor and symptom control in comparison to other more protracted RT regimens. Although previous reports have described clinical complete responses after the QUAD shot radiation regimen, for example Gamez et al., when combining QUAD shot with radiosensitizing chemotherapy [25], no previous reports have shown a pCR when employing neoadjuvant low-dose RT alone. In a study by Fang et al., patients with unresectable T4b oral cavity SCCa underwent preoperative intensity modulated radiation therapy to a radical dose of 66–76 Gy, with 81.8% of patients showing tumor down-grading and 16.7% achieving pCR. ^23^ However, the present case indicates that lower doses of RT, namely Quad-Shot, may provide significant tumor response with useful prognostic information at the time of surgery. For this patient, preoperative Quad-Shot also removed the need for high dose adjuvant therapy with or without chemotherapy which would likely have been recommended had surgery been the initial step in treatment. These implications warrant further study of the use of preoperative Quad-Shot for advanced oral cavity SCCa as a window of opportunity intervention when system issues introduce significant delays between diagnosis and treatment initiation (i.e. surgery).

In summary, this is the first reported case of pCR following treatment of advanced oral cavity SCCa with a single cycle of neoadjuvant Quad-Shot palliative RT. Of particular interest in this patient is the potential benefit of HAART in addition to low dose RT preoperatively. HAART along with chemotherapy and radiation has demonstrated promising results in both solid and blood tumors. This report suggests that continued research into HAART as a cancer therapeutic in both HIV and non-HIV patients is warranted.

## Data Availability

Not applicable.

## Abbreviations

SCCa: Squamous cell carcinoma
RT: Radiation therapy
pCR: Pathologic complete response
HIV: Human immunodeficiency virus
HAART: Highly active antiretroviral therapy
